# Redox homeostasis under summer stress: targeted ROS modulation by foliar kaolin–silicon mixtures in *Vitis vinifera* L

**DOI:** 10.3389/fpls.2026.1855749

**Published:** 2026-07-10

**Authors:** Lia Dinis, Zélia Branco, Renata Moura, Márcia Carvalho, Isaura Castro, Ana Monteiro, Luis Félix, José Moutinho-Pereira, Miguel Baltazar, Sandra Pereira

**Affiliations:** Centre for Research and Technology of Agro-Environmental and Biological Sciences (CITAB), Inov4Agro, University of Trás-os-Montes and Alto Douro (UTAD), Vila Real, Portugal

**Keywords:** antioxidant enzymes, climate change adaptation, kaolin particle films, reactive oxygen species, redox homeostasis, silicon foliar application, summer stress

## Abstract

**Introduction:**

Climate change–driven increases in heat and drought intensity pose major challenges to grapevine performance, largely through the exacerbation of oxidative stress. This study investigated the effects of combined foliar applications of kaolin (Kl) and silicon (Si) on oxidative stress regulation in *Vitis vinifera* L. cv. Touriga Franca grafted onto 1103P under field conditions in the Douro Demarcated Region during the 2023 and 2024 growing seasons.

**Methods:**

Grapevines were treated with different Kl + Si formulations (MiKS), consisting of 2% Kl combined with 2–8% Si, and evaluated at veraison (E-L35) and harvest (E-L38). Leaf proline content, total and specific reactive oxygen species (ROS, H_2_O_2_ and O_2_-), antioxidant enzyme activities (superoxide dismutase- SOD and catalase- CAT), and the expression of key oxidative stress–related genes (FeSOD and CAT) were analyzed.

**Results:**

MiKS formulations significantly modulated grapevine redox metabolism, although responses depended on season, phenological stage, and Si concentration. Intermediate formulations, particularly MiKS 2 (2% Kl + 4% Si) and MiKS 3 (2% Kl + 6% Si), consistently reduced total ROS accumulation by up to 61.7% and decreased H2O2 levels under summer stress conditions. These responses were associated with transient increases in proline accumulation at veraison, enhanced CAT activity, and strong upregulation of *FeSOD* and *CAT* expression, indicating coordinated activation of antioxidant and osmoprotective mechanisms. Although O2•- levels increased in some treatments, this pattern likely reflected controlled redox signaling rather than oxidative damage, as ROS detoxification remained effective. Correlation analysis further supported the close association between ROS modulation and antioxidant regulation.

**Conclusions:**

The combined application of kaolin and silicon, especially at intermediate Si concentrations, promoted a more balanced redox state and improved oxidative stress management under Mediterranean summer conditions. These findings provide mechanistic evidence supporting MiKS formulations as sustainable, low-impact, climate-adaptive tools to enhance grapevine resilience under increasingly hot and dry viticultural scenarios.

## Highlights

- Grapevine redox homeostasis is modulated by MiKS formulations under summer stress
- Responses to MiKS were strongly shaped by interannual climatic variability
- Proline and ROS dynamics reflect stress perception and acclimation capacity
- Kaolin–silicon combinations balance ROS attenuation and redox signalling
- FeSOD and CAT regulation supports improved oxidative stress tolerance

## 1 Introduction

Plants are constantly exposed to a wide range of abiotic stresses, particularly drought and heat, whose effects are further intensified by global warming, prolonged heatwaves, and increasingly erratic precipitation regimes. These stresses disrupt cellular homeostasis and lead to the generation of reactive oxygen species (ROS) (Hasanuzzaman et al., 2020). ROS are oxygen-derived molecules, including superoxide (O_2_•^-^), hydroxyl radicals (•OH), singlet oxygen (¹O_2_), and hydrogen peroxide (H_2_O_2_), that play important roles in plant stress responses (Mhamdi et al., 2010). Among them, H_2_O_2_ is considered a relatively stable signaling molecule involved in regulating several physiological and defense processes (Foyer and Noctor, 2000; Mittler et al., 2004; Sagi and Fluhr, 2006). In plant cells, ROS are mainly generated in chloroplasts, mitochondria, peroxisomes, and at the plasma membrane, while their accumulation is tightly regulated by antioxidant systems to maintain cellular redox homeostasis (Del Río et al., 2006; Corpas et al., 2008; Foyer et al., 2009). Excessive ROS accumulation, however, can lead to oxidative damage and impair cellular functions (Smirnoff and Arnaud, 2019).

To counteract oxidative stress, plants activate enzymatic antioxidant mechanisms, particularly superoxide dismutase (SOD) and catalase (CAT). SOD catalyzes the conversion of superoxide radicals into H_2_O_2_ and O_2_, whereas CAT subsequently decomposes H_2_O_2_ into water and oxygen, thereby limiting oxidative damage (Hansen et al., 2008; Hao et al., 2015). Increased SOD and CAT activities are commonly associated with improved tolerance to drought and heat stress, contributing to the maintenance of photosynthetic performance and redox balance under adverse environmental conditions (Mhamdi et al., 2010; Rahman et al., 2023; Petoumenou and Liava, 2025).

Developing adaptive strategies and protective treatments has become essential for mitigating the impacts of climate change and sustaining crop performance and resilience under increasingly adverse environmental conditions (Terán et al., 2024).

Kaolin is a white, inert clay mineral commonly used as the base for developing particle films applied to plant foliage to mitigate the harmful effects of heat and light stress on plant physiology, productivity, and product quality (Glenn and Puterka, 2004). Several authors have reported that these films reduce thermal and radiation stress by increasing the reflectance of infrared and ultraviolet radiation from the leaf surface, thereby lowering leaf temperature and improving plant water status (Glenn and Puterka, 2004; Dinis et al., 2016b). In the study conducted by Dinis and collaborators, these beneficial effects were observed following the application of 5% Kl. In the work developed by (Bernardo et al., 2017), 5% Kl exogenous application also boosts the antioxidant defense systems in grapevines exposed to summer stress, by increasing enzymatic activities in fruits, which leads to a decrease in ROS production and oxidative damage. A reduction of ROS in berries and leaves was also observed in a work developed by (Dinis et al., 2016a), after the foliar application of 5% Kl. Similar reductions in H_2_O_2_ accumulation and oxidative stress markers following Kl application under drought conditions were also recently reported in other Mediterranean-type crops (Faghihi et al., 2025).

Previous studies have demonstrated that exogenous silicon (Si) (0, 1 and 3 mmol L^− 1^) enhances plant tolerance to drought stress by strengthening the antioxidant defense system, particularly through the stimulation of antioxidant enzymes (Vieira-Filho and Monteiro, 2022; Dinis et al., 2024). Additionally, Shi and collaborators reported that Si application (2.5 mM) in tomato plants reduced ROS accumulation while simultaneously increasing the activities of key antioxidant enzymes, including SOD and CAT, thereby contributing to improved oxidative stress protection (Shi et al., 2016). Similarly, Si application reduced oxidative damage, ROS accumulation, and phenolic content, while enhancing SOD and CAT activities under drought conditions in both wheat and maize. In wheat, positive effects were reported with Si concentrations of 2.11 mmol and 1.0 mM (Gong et al., 2005; Xu et al., 2017), whereas in maize beneficial responses were observed with applications of 4 and 6 mM, as well as 100 mg Si (Parveen et al., 2019; Sattar et al., 2020). Recent evidence also demonstrated that combined kaolin–silicon applications improve grapevine gas exchange, leaf water status, hormonal regulation and anatomical protection under summer stress conditions (Pereira et al., 2025b).

Therefore, this study aims to evaluate the effects of combined foliar applications of different Kl and Si formulations on key markers of oxidative stress responses under challenging environmental conditions. Specifically, the levels of total and specific ROS and proline content, along with the gene expression and enzymatic activity of SOD and CAT, were assessed in grapevines treated with various Kl+Si mixtures, with Kl fixed at 2% and Si ranging from 2 to 8%. The concentrations used in this study were defined based on previous works (Dinis et al., 2016a, Dinis et al., 2016b; Bernardo et al., 2017), with the Kl concentration intentionally reduced from the conventional 5% to 2%, as it was applied in combination with Si, in order to evaluate their combined effects while avoiding excessive leaf surface coverage, whereas Si concentrations were selected according to (Dinis et al., 2024). We hypothesized that the combined application of Kl and Si would enhance the antioxidant defense system and improve redox homeostasis by modulating ROS dynamics, osmoprotective metabolism and antioxidant enzyme regulation, with responses depending on the specific formulation. This work builds on a broader experimental framework designed to assess complementary aspects of grapevine responses to mineral-based foliar treatments under Mediterranean summer conditions. In a companion study using the same vineyard, cultivar, seasons and foliar treatments, Pereira and collaborators addressed canopy gas exchange, vine water status and hormonal dynamics (Pereira et al., 2025b). The present manuscript examines a distinct and non-overlapping set of responses, focusing on leaf redox metabolism through the quantification of total and species-specific ROS, proline accumulation, SOD and CAT activities, and *FeSOD* and *CAT* expression across two years and two phenological stages. In addition, the silicon concentration gradient evaluated here, ranging from 2 to 8%, provides new information on formulation-dependent redox regulation. Together, these data provide mechanistic insight into how mineral-based foliar treatments influence oxidative stress regulation in perennial crops, contributing to the development of sustainable strategies to improve plant resilience under increasing heat and drought stress associated with climate change.

## 2 Materials and methods

### 2.1 Experimental design and sample collection

**Figure 1:**
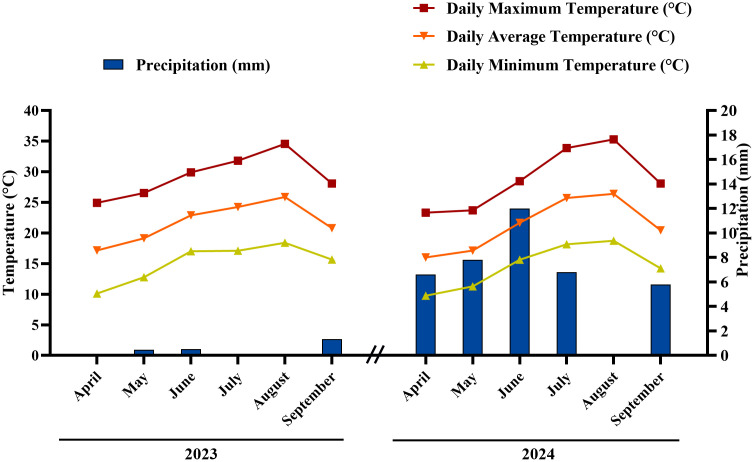
Monthly precipitation (mm) and air temperature (minimum, average and maximum) (°C) of the experimental site, from April to September 2023 and April to September 2024.

The experiment was carried out in the commercial vineyard Quinta de Ventozelo (41°18.954′ N, 8°38.940′ W), located in Ervedosa do Douro, within the Cima Corgo sub-region. This area is characterized by a moderate Mediterranean climate, with hot, dry summers and mild, wet winters, conditions that are particularly favorable for the production of high-quality Port and Douro wines. Meteorological data (precipitation and minimum, average, and maximum temperatures) during the experimental period were recorded by a weather station located within the vineyard and are presented in **Figure 1**.

The experiment was carried out during the 2023 and 2024 growing seasons in a vineyard planted in 2014 with the ‘Touriga Franca’ variety, grafted onto 1103P rootstock.

The experiment followed a randomized complete block design with three blocks. Within each block, all treatments were randomized and applied to one plot per treatment. Each plot consisted of 15 contiguous vines, with at least one guard row between adjacent treatment plots to minimize spray drift. For each treatment × block, three leaves were randomly collected at E-L35 (veraison) and E-L38 (harvest) from different vines within the plot, immediately frozen in liquid nitrogen, and processed as independent biological replicates. All laboratory measurements were performed in technical triplicate and normalized to fresh weight or protein content as described.

In the first year, four treatments were evaluated: untreated grapevines (control group) and grapevines with foliar applications of three different silicon (Si) and kaolin (Kl) formulations. Each formulation contained 2% Kl (Humigel kaolin), with varying Si concentrations (Humigel PlusA) ranging from 2% to 6% (MiKS 1- 2% Kl + 2% Si; MiKS 2- 2% Kl + 4% Si; MiKS 3- 2% Kl + 6% Si). The concentrations used in this study were defined based on previous works (Dinis et al., 2016a, Dinis et al., 2016b; Bernardo et al., 2017), with the kaolin concentration intentionally reduced from the conventional 5% to 2%, as it was applied in combination with silicon, in order to evaluate their combined effects while avoiding excessive leaf surface coverage, while Si concentrations were selected based on previous studies (Dinis et al., 2024). Spray solutions were prepared by first diluting the Si formulation in water, followed by gradual addition of Kl under continuous agitation to obtain a homogeneous suspension; no adjuvants were used. Foliar applications were performed twice per season: the first at bunch closure (E-L31) and the second 15 days later. Sprays were applied in the early morning, using a manual backpack sprayer equipped with a hollow-cone nozzle, operating at 2.0–2.5 bar to ensure fine coverage. Both sides of the canopy were treated to runoff. The application volume averaged 400–600 L ha^−1^ (approximately 0.7–1.0 L vine^−1^), adjusted to canopy size and phenological stage to achieve uniform film formation.

Based on year-1 outcomes, MiKS 1 (2% Kl + 2% Si) displayed limited and stage-dependent effects, with no consistent multi-parameter improvements relative to the control across ROS, enzyme activities, and gene expression (with the exception of a punctual effect on proline at E-L38). To strengthen inference on dose–response and focus on formulations with clearer, cross-trait benefits, MiKS 1 was discontinued and a higher Si level (MiKS 4: 2% Kl + 8% Si) was introduced, expanding the Si gradient to 2–8%.

### 2.2 Proline determination

To determine proline, 200 mg of each sample was extracted in 1 ml of 3% SSA (sulfosalicylic acid), following the method described by Bernardo and collaborators (Bernardo et al., 2022).The extracts were homogenized in 3 cycles of ultrasound (15 sec), ice and vortex, and after centrifuged at 1,368 x g for 20 minutes at 4 °C. Next, to each supernatant sample was added glacial acetic acid 17.4 M and ninhydrin 12.5 g 50 mL^-1^ and heated at 100 °C in a water bath for 60 minutes. After waiting until it cools down on ice and added 1 ml of toluene and mixed on vortex. Absorbance was measured at 520 nm, on a spectrophotometer (SPECTRUM star Nano, BMG Labtech GmbH, Germany). A proline standard curve was used for quantification, and results were expressed as mg g^-1^ FW.

### 2.3 Protein quantification

The total protein concentration in the leaf extracts was determined by the Bradford method, using bovine serum albumin (BSA) as a standard, as described by (Pereira et al., 2025b). The results were used to normalize enzyme activity.

### 2.4 Quantification of reactive oxygen species

The accumulation of reactive oxygen species (ROS) was quantified using 2′,7′-dichlorodihydrofluorescein diacetate (DCFH-DA), following the methodology described by (Jambunathan, 2010; Kong et al., 2013), with slight modifications. In 96-well plates, 10 µL of extract were mixed with 100 µL of Tris-HCl buffer (10 mM, pH 7.4) and 10 µL of DCFH-DA 10 mg mL^-1^ (Sigma–Aldrich, Germany). The mixtures were incubated at 25 °C for 20 min in the dark. The fluorescence of the oxidized product (DCF) was measured using a spectrofluorometer (Varian Cary Eclipse, Varian, Palo Alto, USA) equipped with a microplate reader, with excitation and emission wavelengths set at 485 and 530 nm, respectively. All samples were analyzed in triplicate and corrected using a blank. The quantification was performed based on a DCF standard curve, and results were expressed as nM DCF mg^-1^ protein.

### 2.5 Hydrogen peroxide (H_2_O_2_) quantification

To quantify the H_2_O_2,_ 25 mg of fresh leaf sample and 1 mL of 1% TCA (Trichloroacetic acid) were used (Velikova et al., 2000; Junglee et al., 2014). After three cycles consisting of 15 s ultrasound treatment followed by cooling on ice, the samples were centrifuged at 12,000 x g for 15 minutes at 4 °C. In a microplate, 50 µL of potassium phosphate buffer (100 mM; pH 7) and 100 µL of potassium iodine 1 M (KI) were added to 50 µL of supernatant, incubated for 5 min, and the absorbance was read at 350 nm. A calibration curve was prepared using a 30% H_2_O_2_ stock solution, with concentrations ranging from 3.90625 µM to 1000 µM, and the results expressed as µmol H_2_O_2_·g^-1^ FW.

### 2.6 Superoxide (O_2_•^-^) quantification

Superoxide concentration was measured following the method described by several authors (Li et al., 2018; Pinto et al., 2023), with minor adjustments. 150 mg of fresh leaf sample were homogenized in potassium phosphate (PK) buffer (65 mM; pH 7.8), containing 1% polyvinylpolypyrrolidone (PVPP), and centrifuged at 5,000 x g for 20 minutes at 4°C. For the quantification, in a microplate, 45 µL of supernatant, 45 µL of PK buffer, and 100 µL of hydroxylamine hydrochloride were added, and incubated at 25 °C for 20 minutes. After this, 100 µL of 3-aminobenzenesulphonic acid and 100 µL of 1-naphtylamine were added, and samples were incubated for 30 minutes, before reading the absorbance at 530 nm. A calibration curve was prepared using a sodium nitrite (NaNO_2_) solution, with concentrations ranging from 0.003906 to 1 µmol ml^-1^. The superoxide was calculated according to a standard curve, and the results expressed as O_2_•^-^ µmol·g^-1^ FW.

### 2.7 Determination of antioxidant enzymatic activities

#### 2.7.1 Superoxide dismutase quantification

Superoxide dismutase was determined using the Superoxide Dismutase Colorimetric Activity Kit (Thermo Fisher Scientific, Cat. No. EIASODC), according to the manufacturer’s instructions. For this, 100 mg of sample was weighed and homogenized on ice with 1 mL of PBS buffer (pH 7.4), centrifuged at 10,000 x g for 15 minutes at 4 °C. The supernatant was collected and diluted 1:4 with Assay Buffer. The sample and substrate (1x) were added to a 96-well microplate and read at 450 nm. Xanthine oxidase (1x) was then added to the wells of the plate, and the reaction was conducted at room temperature for 20 minutes, followed by a new reading at the same wavelength. A calibration curve was prepared using the SOD standard provided in the kit, with concentrations ranging from 0 to 92 U·mL^-1^. Enzyme activity was calculated from the standard curve and expressed in SOD U·mg^-1^ of protein.

#### 2.7.2 Catalase activity quantification

For the preparation of enzyme extracts and catalase determination, the Catalase Colorimetric Activity Kit (Thermo Fisher Scientific, Cat. NO EIACATC) was used, following the instructions provided. For the enzyme extracts, 100 mg of leaf tissue was weighed and 1 mL of Assay buffer (1x) was added, homogenized on ice and centrifuged at 10,000 x g for 15 minutes at 4°C. The supernatant and hydrogen peroxide were added to a microplate and incubated for 30 minutes at room temperature. Next, substrate and colorimetric reagent (HRP) were added to the same wells, and another incubation was performed for 15 minutes at room temperature. The reading was performed at a wavelength of 520 nm. A calibration curve was prepared using the standard solutions provided in the kit, allowing the enzyme activity in the grapevine leaf samples to be quantified. The results were expressed in CAT U·mg^-1^ of protein.

### 2.8 Total RNA extraction, cDNA synthesis and RT-qPCR

Leaves collected at E-L35 (*veraison*) and E-L38 (maturation) development stage were collected in three independent biological replicates. Total RNA from 80 mg of leaf tissue was extracted using NZY Plant/Fungi RNA Isolation kit (NZYtech, Lisbon, Portugal), following the manufacture’s protocol. RNA integrity and concentration were estimated using the A_260_/A_280_ ratio through the spectrophotometer Nanodrop One (Thermo Fisher Scientific, Waltham, MA USA) and 1% agarose gel electrophoresis.

Per each sample, total RNA (500 ng) reverse transcription was prepared using NZY First-Strand cDNA Synthesis kit (NZYtech, Lisbon, Portugal), following the manufacturer’s procedure. The differential gene expression of three oxidative stress-related genes involved in ROS metabolism (*FeSOD, Cu/ZnSOD* and *CAT*; **Table 1**) was analyzed by semi-quantitative RT-PCR. Subsequently, the two genes presenting the highest differential expression (*FeSOD* and *CAT*) were further evaluated by real-time RT-PCR using StepOnePlus Real Time PCR (Applied Biosystems, Foster City, USA) according to the MIQE criteria (Minimum Information for the Publication of Quantitative Real-Time PCR Experiments) (Bustin et al., 2009). Each reaction (10 µL) contained 500 nM of each primer, 2 µL of cDNA (1:5 dilution), 5 µL of NZYSupreme qPCR Green Master Mix (2x) ROX (NZYtech, Lisbon, Portugal). Thermal cycling conditions were a hold stage at 95 °C for 5 min, followed by 40 cycles: 95 °C for 5 s and 60 °C for 25 s. A melting cycle with temperature ranging from 60 to 95 °C was introduced to detect non-specific amplification in cDNA samples. Each one of the three biological replicates were used in three technical replicates. Gene transcripts were quantified upon normalization to one reference gene *(Elongation factor 1-alfa*; *EF1α*) (Ferreira et al., 2019) by comparing the threshold cycle (Ct) of each target gene with the reference gene Ct. The relative quantification per each gene was calculated according to the 2^−ΔΔCt^ method, where ΔCt is the difference in threshold cycle between the geometric means of the target and reference gene (EF1α) and ΔΔCt is the difference between the average ΔCt of the target and control samples. Only threshold quantification cycle (Ct) values, leading to a Ct mean with a standard deviation below 0.5, were considered. Mean PCR efficiency per gene was estimated using standard curves based on fivefold dilutions of corresponding cDNA mix of all the samples (in triplicate). The efficiency values varied from 96 to 108% for reference and target genes and were determined and calculated by the StepOnePlus Real Time PCR software (Applied Biosystems, Foster City, USA).

**Table 1:** Primer sequences, and annealing temperatures for the genes analyzed.

Gene	Primer sequence (5’ – 3’)	Reference	GeneBank accession number
*EF1α* (*Elongation factor 1-alpha*)	F: GAACTGGGTGCTTGATAGGC R: AACCAAAATATCCGGAGTAAAAGA	(Gutha et al., 2010)	GU585871
*CAT* (*Catalase*)	F: GGTGTTCACACCTTCACTCT R: GAGATCCTGAGTAGCATGACTG	(Singh et al., 2019)	–
*Fe_SOD* (*Superoxide dismutase*)	F: CCTTTGTGAACCTAGGCGAACC R: TGGCCGGGTTAGCTTGAACTC	(Singh et al., 2019)	-
*Cu/Zn_SOD* (*Superoxide dismutase*)	F: AGATTGGCATGTGGTGTTGTTG R: ACTCCCACATTACCCAACAACA	(Singh et al., 2019)	–

### 2.9 Statistical analyses

Data analysis was performed using the SPSS 20.0 software (SPSS Software, Chicago, IL, USA). After testing for analysis of variance (ANOVA) assumptions, statistical differences among treatments within each developmental stage and year were evaluated by one-way factorial ANOVA, followed by the *post hoc* Tukey test. Different letters represent significant differences (*p* < 0.05) among the applied formulations. A heat map was also constructed based on Pearson correlations across all variables.

## 3 Results

The effects of the different MiKS foliar formulations on proline content and on the accumulation of reactive oxygen species and the activity of the antioxidant enzymes SOD and CAT were evaluated across two consecutive seasons (2023 and 2024) and at two phenological stages (E-L35 and E-L38), allowing for an integrated assessment of the plants’ redox status under each treatment.

### 3.1 Climate conditions

In 2023, average temperatures rose steadily from ~17 °C in April to 26 °C in August, before declining to 8 °C by December. Minimum and maximum values followed similar patterns, ranging from 10 °C to 18 °C and from 20 °C to 35 °C, respectively. Precipitation was consistently below average, with virtually no rainfall between July and August, suggesting potential water stress that could have influenced grapevine physiology and irrigation needs.

In contrast, 2024 was considerably wetter. Average temperatures increased from ~9 °C in January to 26 °C in August, before falling to 20 °C in September. Minimum and maximum temperatures varied between 6 °C and 19 °C, and 13 °C and 35 °C, respectively. The highest rainfall was recorded in March (~19 mm), while no precipitation occurred in August. Compared with 2023, the higher rainfall and milder dry periods in 2024 indicate less severe drought conditions.

### 3.2 Proline synthesis

**Figure 2:**
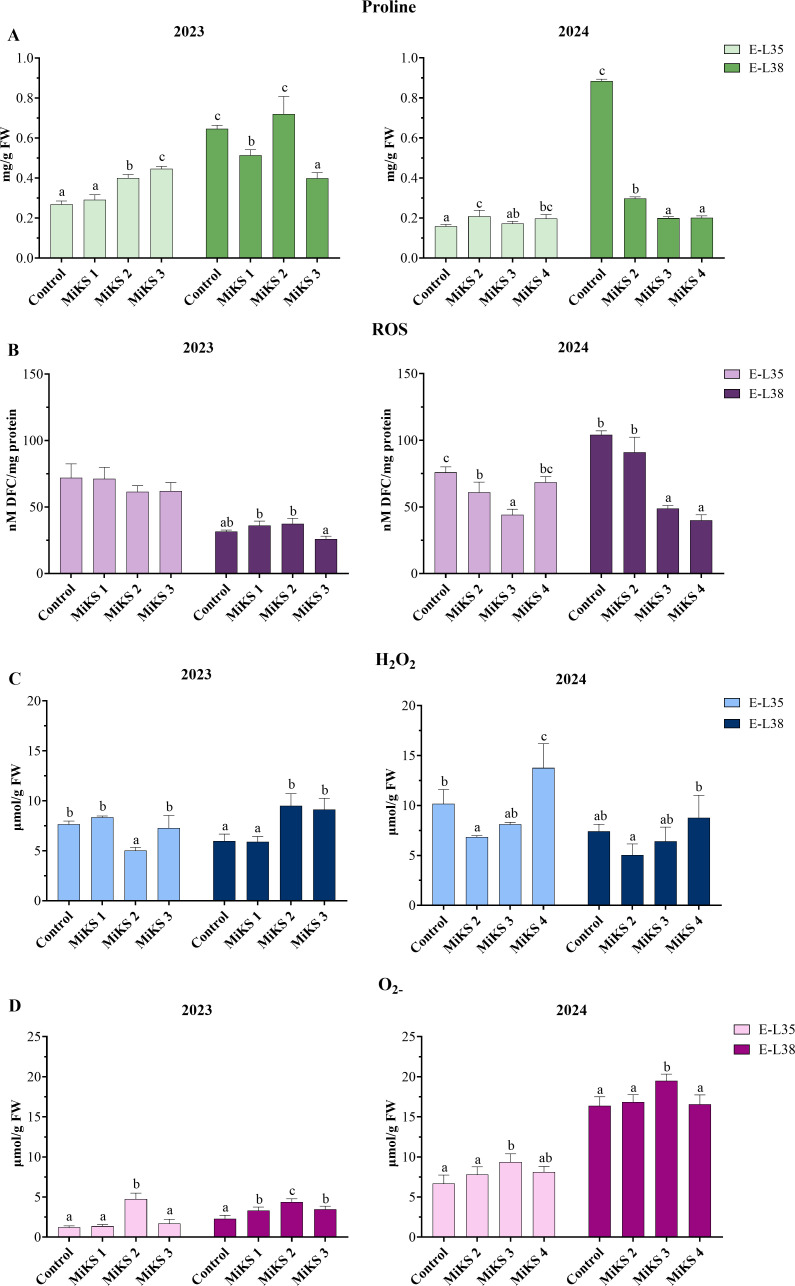
**(A)** Leaf proline concentration (mg proline/g FW); **(B)** Leaf reactive oxygen species (ROS) concentration (nM DFC/mg protein); **(C)** Leaf hydrogen peroxide (H_2_O_2_) concentration (µmol/g FW) and **(D)** Leaf superoxide (O_2_•^-^) concentration (µmol/g FW), under different foliar treatments: control (untreated); MiKS 1 (Kl 2% + Si 2%); MiKS 2 (Kl 2% + Si 4%); MiKS 3 (Kl 2% + Si 6%); MiKS 4 (Kl 2% + Si 8%). Results are expressed as mean ± standard deviation. Different letters indicate significant differences (*p* < 0.05) and the absence of letters indicates no statistically significant differences (*p* > 0.05) between treatments, according to Tukey’s test.

Results from the quantification of proline are presented in **Figure 2A**. At E-L35 in 2023, significant differences were detected among treatments. MiKS 2 and MiKS 3 showed increases of 49.3% and 66.3% relative to the control, and proline concentration in MiKS 3 was 11.4% higher than in MiKS 2. At E-L38, MiKS 2 exhibited the highest proline levels, although not significantly different from the control, but 40.1% higher than MiKS 1 and 80.3% higher than MiKS 3. Conversely, MiKS 1 and MiKS 3 displayed significantly lower proline contents than untreated plants, with decreases of 20.4% and 38.2%, respectively. These two treatments also differed from each other, with MiKS 1 showing 28.7% higher proline concentration than MiKS 3. In 2024, at E-L35, only MiKS 2 and MiKS 4 differed significantly from the control, showing increases of 31.2% and 23.9%, respectively. Proline content in MiKS 2 was also 21.1% significantly higher than in MiKS 3. Finally, at E-L38 in 2024, the control presented the highest proline concentration, while all foliar treatments showed significant reductions of 66.3% in MiKS 2, 77.5% in MiKS 3, and 77.3% in MiKS 4. MiKS 3 and MiKS 4 also showed significant reductions of 33.1% and 32.6%, respectively, when compared with MiKS 2.

### 3.3 Leaf total and individual ROS modulation

Results from the quantification of total ROS are presented in **Figure 2B**. In 2023, at E-L35, no significant differences were observed among treatments. However, at E-L38, MiKS 3-treated plants exhibited the lowest ROS concentration, showing significantly lower values than MiKS 1 and MiKS 2 by 27.9% and 30.6%, respectively. In 2024, results at E-L35 revealed that control plants had the highest concentration of ROS, with MiKS 2 and MiKS 3-treatead plants showing significant reductions of 19.7% and 41.8%, respectively, in comparison. Furthermore, among MiKS treatments, ROS values in MiKS 3 were also 27.6% significantly lower than in MiKS 2, and by 35.6% in MiKS 4. A similar trend was observed at E-L38, where leaves from untreated plants again showed the highest ROS concentration, followed by MiKS 2, while MiKS 3 and MiKS 4 recorded significant reductions of 53.0% and 61.7%, respectively, relative to the control.

The results of the quantification of hydrogen peroxide (H_2_O_2_) are represented in **Figure 2C**. At E-L35 in 2023, MiKS 2-treated plants exhibited the lowest of this radical, being significantly lower than the control by 34.4%, than MiKS 1 by 40.0%, and than MiKS 3 by 31.0%. At E-L38 of the same year, both the control and MiKS 1 showed similar values, whereas MiKS 2 and MiKS 3 displayed significantly higher H_2_O_2_ concentrations increasing by 58.7% and 52.4%, respectively, compared with the control. In 2024, the quantification of H_2_O_2_ also revealed distinct responses among treatments. For instance, all MiKS treatments differed from the control at E-L35, with H_2_O_2_ concentration decreasing by 32.6% and 20.0% in MiKS 2 and MiKS 3 plants respectively, while increasing by 35.4% in MiKS 4 ones. Moreover, MiKS 2 and MiKS 3 also differed significantly from MiKS 4, showing 50.2% and 40.9% lower H_2_O_2_ concentrations, respectively. Finally, at E-L38 in 2024, significant differences were only observed between MiKS 2 and MiKS 4 plants, with MiKS 2-treated ones exhibiting the lowest content and being 42.6% lower than those of MiKS 4.

Result from superoxide (O_2_•^-^) are shown in **Figure 2D**. At E-L35 in 2023, the highest concentration was observed in plants treated with MiKS 2, increasing significantly in comparison to the control (287.1%), MiKS 1 (247.4%), and MiKS 3 (178.0%). At E-L38 of the same year, the untreated plants displayed the lowest O_2_•^-^, which increased significantly in MiKS 1-plants by 43.9%, MiKS 2 by 90.4%, and MiKS 3 by 51.3%. Among the MiKS treatments, MiKS 2 also presented the highest levels, while MiKS 1 and MiKS 3 were 24.5% and 20.5% lower, respectively. In 2024, at E-L35, the highest O_2_•^-^ concentration was observed in MiKS 3-treated plants, being significantly higher than the control (40.1% increase) and MiKS 2 (19.9% increase). This pattern persisted at E-L38, where MiKS 3 plants surpassed the control ones by 19.0%, MiKS 2 by 15.7%, and MiKS 4 by 17.8%.

### 3.4 Antioxidant enzymatic activity and gene expression

**Figure 3:**
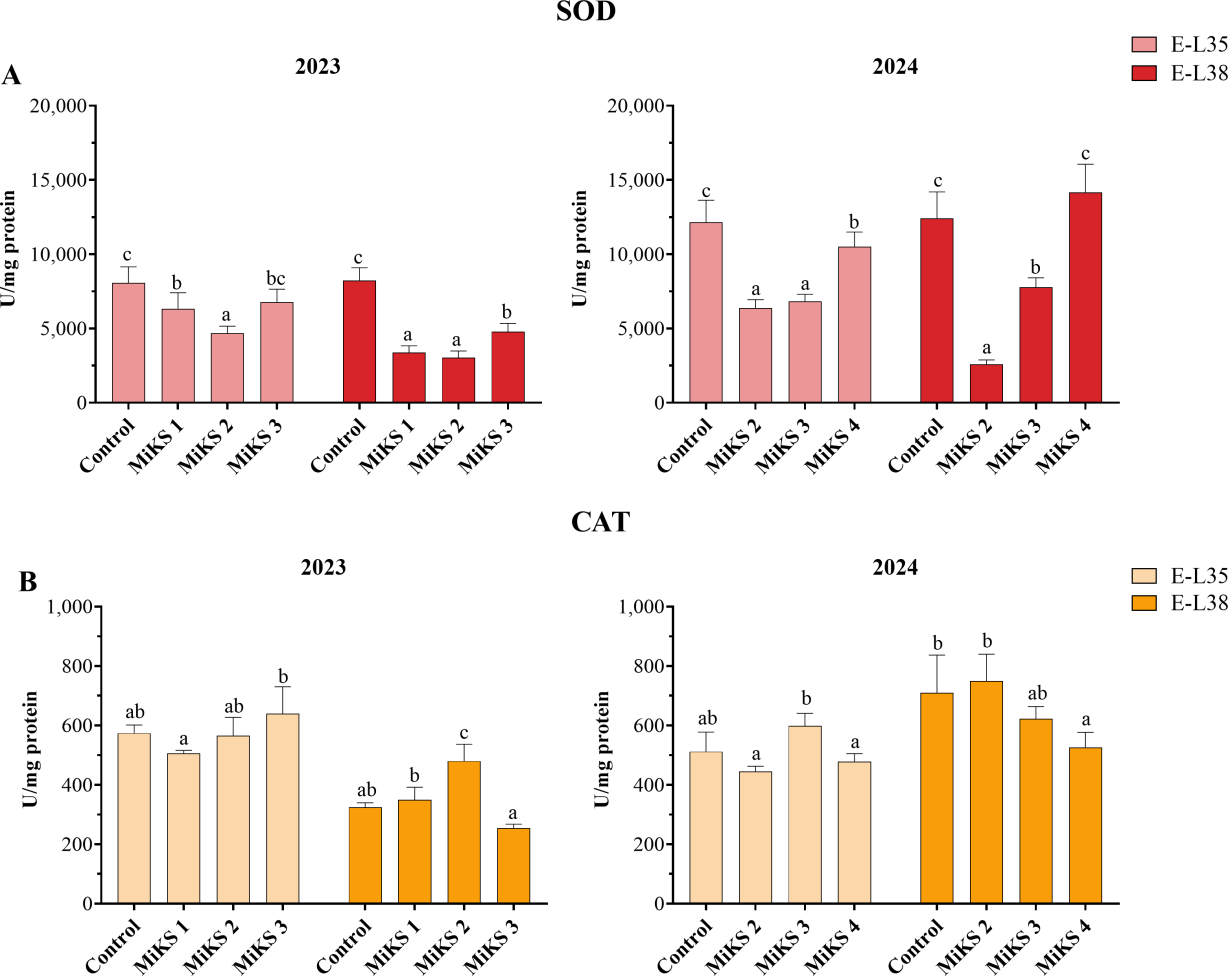
**(A)** Leaf superoxide dismutase (SOD) activity (U/mg protein) and **(B)** Leaf catalase (CAT) activity (U/mg protein) after different foliar treatments: control (untreated); MiKS 1 (Kl 2% + Si 2%)*;* MiKS 2 (Kl 2% + Si 4%); MiKS 3 (Kl 2% + Si 6%); MiKS 4 (Kl 2% + Si 8%). Results are expressed as mean ± standard deviation. Different letters indicate significant differences (*p* < 0.05) and the absence of letters indicates no statistically significant differences (*p* > 0.05) between treatments, according to Tukey’s test.

Analysis of SOD activity (**Figure 3A**) showed that, in 2023 at E-L35, the highest enzymatic activity occurred in untreated plants and decreased significantly in MiKS 1 and MiKS 2 by 21.6% and 42.2%, respectively. In addition, MiKS 2 exhibited 30.9% lower activity than MiKS 3. Similarly, to E-L35, at E-L38 the untreated plants exhibited the highest SOD activity, decreasing significantly in the MiKS 3 treatment by 41.9%, while MiKS 1 and MiKS 2 presented even larger reductions of 58.9% and 63.1%, respectively. For the same developmental stage, between MiKS treatments, SOD activity in MiKS 1 and MiKS 2-treated plants was also 29.2% and 36.4% significantly lower than in MiKS 3-treated ones. In 2024, at E-L35, SOD activity again peaked in untreated plants, followed by a progressive decline in the mixture treatments, with decreases being observed for MiKS 2 (47.5%), MiKS 3 (43.8%), and MiKS 4 (13.4%), relative to the control. Between the MiKS treatments, plants sprayed with MiKS 4 displayed the highest SOD activity, exceeding MiKS 2 and MiKS 3 by 65.0% and 54.0%, respectively. Lastly, at E-L38, SOD activity was highest in MiKS 4, differing significantly from MiKS 2 (81.8% decrease) and MiKS 3 (45.2% decrease). Both of these treatments also showed strong reductions compared with the control (−79.2% and −37.4%, respectively), while between themselves, MiKS 2 exhibited 66.8% less SOD activity than MiKS 3.

CAT activity (**Figure 3B**) showed limited treatment effects at E-L35 in 2023, with the only significant difference being between MiKS 1 and MiKS 3, with MiKS 1 displaying 20.8% lower activity than MiKS 3. In the same year at E-L38, MiKS 2–treated plants exhibited the highest CAT activity and were the only group that differed significantly from the control (48.1% increase). This enzymatic activity in MiKS 2 also exceeded MiKS 1 and MiKS 3 by 37.5% and 89.0%, respectively. Between MiKS 1 and MiKS 3 at E-L38, MiKS 3 showed 27.2% lower activity. In 2024, at E-L35, significant differences were observed only among the MiKS formulations. Plants treated with MiKS 3 displayed the highest CAT activity, exceeding MiKS 1 and MiKS 2 by 34.4% and 25.3%, respectively. At E-L38 of the same year, MiKS 4 exhibited the lowest CAT activity, decreasing significantly relative to both the control (26.0%) and MiKS 2 (29.8%), the latter showing the highest activity among treatments.

**Figure 4:**
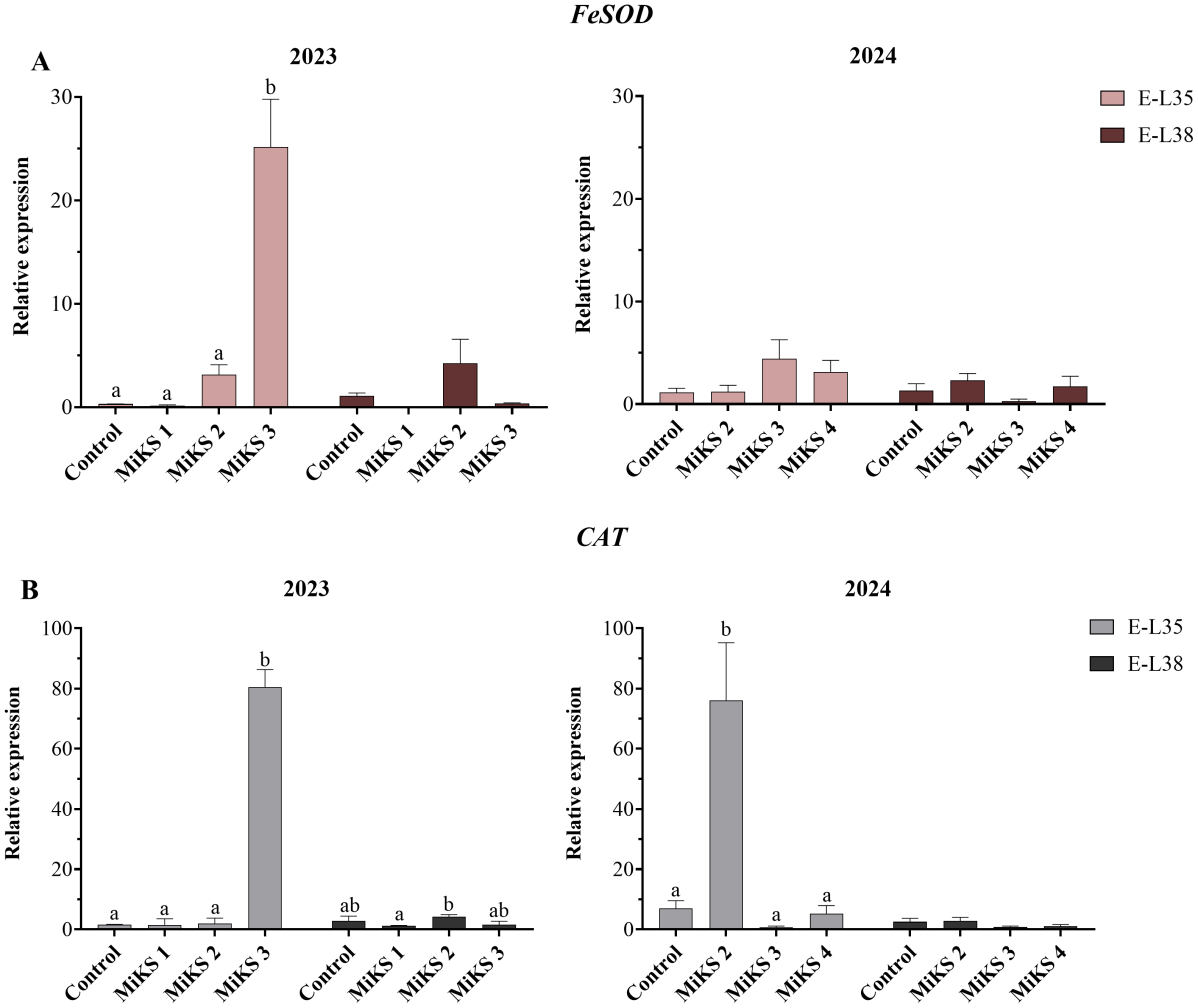
Expression of the *FeSOD* gene **(A)** and *CAT* gene **(B)**, evaluated by qPCR, in the leaves of grapevines under different foliar treatments: control (untreated); MiKS 1 (Kl 2% + Si 2%)*;* MiKS 2 (Kl 2% + Si 4%); MiKS 3 (Kl 2% + Si 6%); MiKS 4 (Kl 2% + Si 8%). The relative expression levels were obtained after normalization with the expression of *EF1-α* reference genes. Values are the mean ± SD (n = 9). Different letters indicate significant differences (*p* < 0.05) and the absence of letters indicates no statistically significant differences (*p* > 0.05) between treatments, according to Tukey’s test.

A total of three oxidative stress related genes (*FeSOD*, *CuSOD*, and *CAT*) were evaluated by semi-quantitative RT-PCR, but significant differences between treatments were only detected in two of them (*FeSOD* and *CAT*). Overall, MiKS treatments upregulated or tended to upregulate the expression of the *FeSOD* gene at both phenological stages and across both years (**Figure 4A**). Nonetheless, significant differences between the treatments for this gene were only observed in the 2023 season at E-L35, with plants from the MiKS 3 treatment presenting the highest *FeSOD* expression comparatively to the control and remaining treatments. At E-L38 of the same year, the highest relative expression levels of *FeSOD* were observed in plants treated with MiKS 2, with no significant differences being observed. In 2024 there were no significant differences between treatments regarding *FeSOD* gene expression. Nevertheless, response patterns within the same phenological stage were largely consistent with those observed in 2023. In fact, at E-L35, MiKS 3-plants exhibited the highest *FeSOD* relative expression and at E-L38, MiKS 2-plants presented the highest values.

In the analysis of the *CAT* gene expression, statistically significant differences among treatments were detected in the 2023 season at both stages, and in 2024 only at E-L35 (**Figure 4B**). During 2023, at E-L35, MiKS 3 showed the highest relative *CAT* expression levels with significant differences to control and remaining treatments and at E-L38, MiKS 2-plants had the highest relative *CAT* expression, significantly different from MiKS 1. Finally, in 2024 at E-L35, MiKS 2-treated plants reached the highest *CAT* relative expression levels. On the other hand, *CAT* expression at E-L38 was very reduced, with no significant differences being observed between treatments.

### 3.5 Correlation analysis of oxidative stress markers, antioxidant enzyme activity, and gene expression

**Figure 5:**
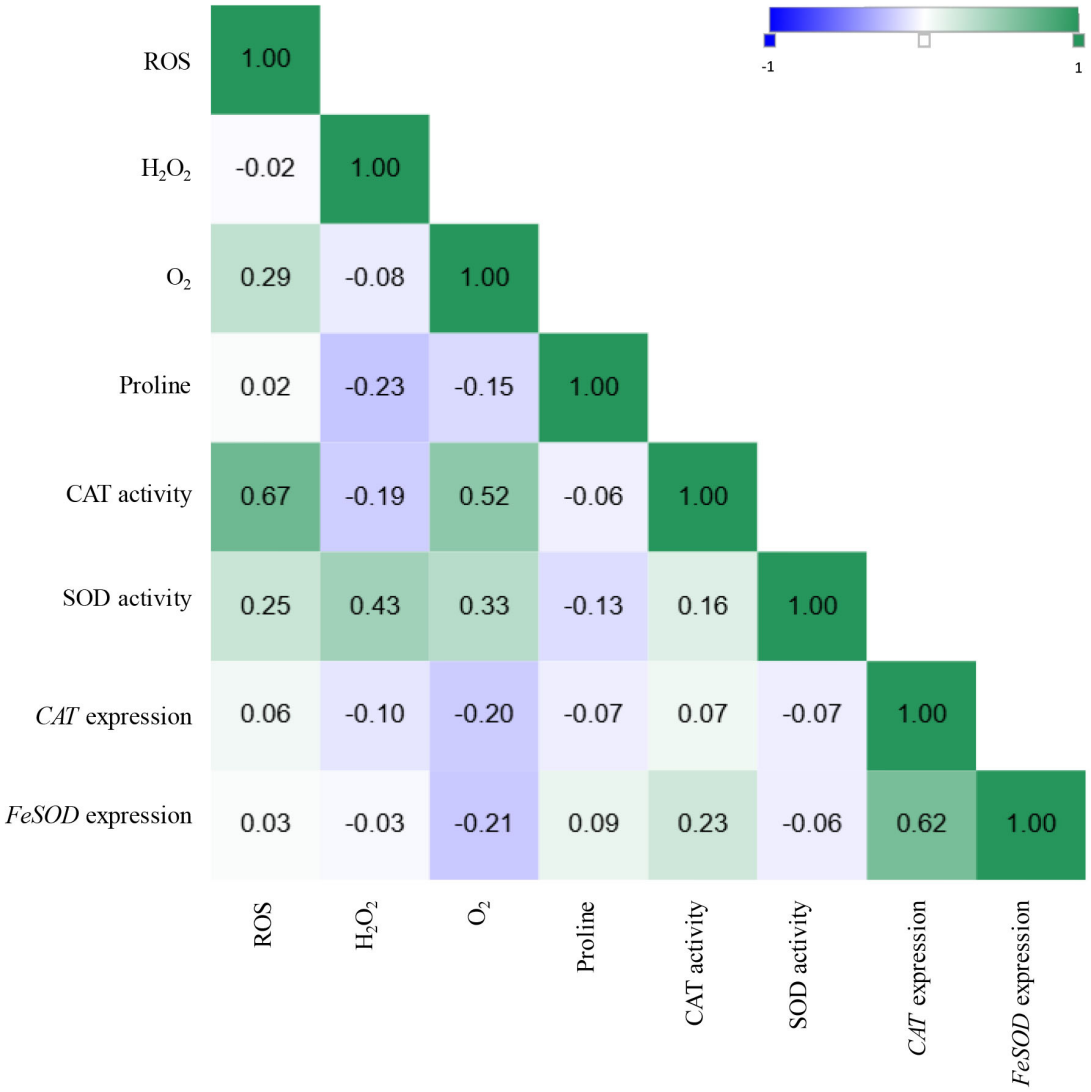
Pearson correlation heatmap illustrating the relationships among total reactive oxygen species (ROS), hydrogen peroxide (H_2_O_2_), superoxide radical (O_2_•^-^), proline content, antioxidant enzyme activities (CAT and SOD), and relative gene expression levels of *CAT* and *FeSOD* in *Vitis vinifera* cv. Touriga Franca under different kaolin–silicon treatments. Positive correlations are represented in green and negative correlations in blue, with color intensity proportional to the correlation coefficient (r).

The correlation analysis (**Figure 5**) revealed distinct relationships among oxidative stress markers, antioxidant enzyme activities, and gene expression levels. ROS showed a strong positive correlation with CAT activity (r = 0.67), while O_2_•^-^ was moderately positively correlated with CAT activity (r = 0.52). H_2_O_2_ presented a moderate positive correlation with SOD activity (r = 0.43). A strong positive association was also observed between CAT and SOD gene expression levels (r = 0.62). In contrast, proline exhibited weak or negative correlations with most evaluated parameters. Gene expression levels of CAT and SOD generally showed low correlations with their respective enzymatic activities.

## 4 Discussion

### 4.1 Seasonal climatic variability and overall plant responses

Climate variability exerts a strong influence on grapevine metabolism and stress responses (Dinis et al., 2014). In the present study, the physiological, biochemical, and molecular differences observed between years were associated with the contrasting climatic conditions recorded during the two growing seasons. The 2023 season was characterized by prolonged summer drought, almost no rainfall during July and August, and sustained high temperatures. Complementary measurements of predawn leaf water potential and stomatal conductance obtained from the same experimental trial (Pereira et al., 2025b) support the occurrence of summer water limitation and treatment-dependent physiological adjustments, consistent with the redox patterns observed here. Nevertheless, because thermal and drought stress were not directly quantified in this study, the observed responses should be interpreted as associations consistent with stress mitigation rather than definitive causal evidence.

Under these conditions, MiKS-treated vines, particularly MiKS 2 and MiKS 3, showed increased proline accumulation and enhanced FeSOD and CAT expression at E-L35, suggesting an early activation of antioxidant and osmoprotective mechanisms. In contrast, the 2024 season experienced higher precipitation and shorter dry periods (**Figure 1**), resulting in milder stress conditions, especially during ripening. Under these conditions, MiKS treatments appeared to act mainly by limiting excessive ROS accumulation rather than triggering strong stress-related metabolic responses. This pattern was reflected by lower ROS and H_2_O_2_ concentrations and reduced proline accumulation at E-L38 in treated plants, whereas control plants exhibited higher proline levels, likely reflecting a stronger stress-related response. The attenuation of antioxidant enzyme activity and gene expression responses in 2024 further suggests that MiKS formulations exerted a stabilizing effect under comparatively moderate environmental pressure.

These interannual differences indicate that MiKS-induced responses are highly dependent on climatic context, being more pronounced under severe summer conditions and more moderate under milder environmental scenarios.

### 4.2 Proline accumulation and osmotic adjustment

Proline metabolism is widely regarded as a sensitive indicator of plant physiological status under abiotic stress (Renzetti et al., 2024), reflecting both stress intensity and the effectiveness of adaptive responses. In this context, the proline dynamics observed in the present study provide valuable insight into the tolerance mechanisms induced by MiKS formulations. The pronounced accumulation of proline under MiKS 2 and MiKS 3 at E-L35 in 2023 is consistent with its well-established role as an osmoprotectant (Renzetti et al., 2024), contributing to the maintenance of leaf water potential, stabilization of proteins and cellular membranes, and mitigation of oxidative damage through ROS scavenging (Szabados and Savouré, 2010; Hayat et al., 2012). Notably, this accumulation coincided with a slight reduction in total ROS levels in treated plants, supporting the view that enhanced proline synthesis contributes to improved redox homeostasis under stress conditions (Renzetti et al., 2024). Similar rapid increases in leaf proline content under heat and drought stress have been widely reported, where proline also functions as a transient nitrogen reservoir and as an osmotic and redox buffer (Verslues and Sharma, 2010; Sharma et al., 2011; Kaur and Asthir, 2015).

Conversely, the marked reduction in proline levels observed at E-L38 in 2024 across all MiKS-treated plants may indicate lower oxidative and osmotic pressure during ripening (Szabados and Savouré, 2010). This decrease likely reflects an improved leaf microclimate and a sustained reduction in oxidative pressure under MiKS treatments, as evidenced by the overall decline in ROS-related parameters. At this phenological stage, proline accumulated during earlier stress periods may be remobilized to support other metabolic demands, including protein synthesis and pathways associated with berry development and maturation (Kishor, 2015). Such remobilization is consistent with the dual role of proline as both a stress-related metabolite and a source of reduced nitrogen and carbon (Kishor, 2015), reinforcing the hypothesis that MiKS formulations may indirectly influence fruit quality through modulation of stress metabolism and resource allocation.

### 4.3 Modulation of ROS accumulation by MiKS formulations

As previously mentioned, foliar MiKS applications effectively modulated leaf redox status throughout both growing seasons. In general, MiKS treatments reduced total ROS accumulation, particularly MiKS 2 and MiKS 3 at E-L35 and MiKS 3 and MiKS 4 at E-L38 in 2024, supporting the hypothesis that the combined application of kaolin and silicon can mitigate oxidative stress under summer stress conditions. These effects are consistent with the capacity of kaolin-based particle films to improve the leaf microclimate by increasing reflectance and reducing incident radiation and leaf temperature (Dinis et al., 2016b; Wang et al., 2022), while silicon may contribute to structural and physiological protection mechanisms (Dinis et al., 2024). The consistency of ROS reductions across phenological stages and growing seasons strengthens the robustness of these responses and suggests that MiKS formulations can provide stable redox modulation under variable environmental conditions.

Previous studies have demonstrated that kaolin particle films reduce sunburn incidence, lipid peroxidation and ROS accumulation by attenuating excess radiation and leaf overheating (Glenn and Puterka, 2004). In grapevine cv. Touriga Nacional, Dinis and collaborators reported increased antioxidant capacity and phenolic accumulation in leaves and berries treated with kaolin, which was interpreted as a consequence of reduced oxidative damage to cellular membranes (Dinis et al., 2016a). Similarly, Wang and collaborators showed that kaolin applications in Cabernet Sauvignon were associated with enhanced activity of antioxidant enzymes such as SOD and CAT, following the initial ROS burst triggered by biotic stress (Wang et al., 2022). Collectively, these studies support the view that the kaolin component of MiKS formulations primarily reduces basal ROS generation by moderating excess light and heat stress, thereby stabilizing photosynthetic function.

In agreement with the present results, a complementary study conducted within the same experimental trial demonstrated that MiKS 3 and MiKS 4 improved gas exchange, stomatal conductance, leaf water status and chlorophyll *a* fluorescence under comparable summer conditions (Pereira et al., 2025b). These effects were attributed to thicker cuticles, enhanced epicuticular wax deposition and increased levels of specific phenolic compounds, such as *ortho*-diphenols, which collectively enhance tolerance to high irradiance and temperature. The convergence between these physiological improvements and the reductions in total ROS observed in the present study can suggest that MiKS formulations act in an integrated manner: by improving the energy and thermal balance at the leaf–atmosphere interface and by indirectly supporting a more efficient antioxidant system, thereby reducing the need for emergency stress responses associated with severe oxidative pressure (Dinis et al., 2016b; Pereira et al., 2025b).

Interestingly, distinct patterns were observed among individual ROS species. While reductions in total ROS were generally associated with lower H_2_O_2_ concentrations, moderate increases in H_2_O_2_ and O_2_•^-^ were detected in some treatments and phenological stages, particularly in MiKS 3-treated plants. This indicates that MiKS formulations may influence not only the magnitude but also the composition of the ROS pool, reflecting a possible shift from oxidative damage toward controlled redox signaling rather than a simple suppression of ROS production (Turkan, 2018).

It is important to emphasize that total ROS and H_2_O_2_ do not necessarily exhibit parallel trends because they represent different components of the cellular redox network and were quantified using different analytical approaches. The DCFH-DA assay provides an integrated estimate of overall oxidative activity derived from multiple ROS species and downstream oxidation products, whereas the KI-based assay specifically quantifies H_2_O_2_ accumulation. Therefore, reductions in total ROS may coexist with stable or moderately increased H_2_O_2_ concentrations if other highly reactive ROS species are efficiently detoxified while H_2_O_2_ is transiently maintained. This distinction is biologically relevant because H_2_O_2_ is not only a marker of oxidative stress, but also a relatively stable signaling molecule involved in stress acclimation and antioxidant regulation. Moderate and transient H_2_O_2_ accumulation has been associated with the activation of antioxidant enzymes, stress-responsive genes, stomatal regulation, and cellular defense pathways (Apel and Hirt, 2004). In the present study, treatments showing lower total ROS together with moderate H_2_O_2_ accumulation also displayed coordinated antioxidant responses, including enhanced CAT activity and/or increased FeSOD and CAT expression, supporting the interpretation of regulated redox signaling rather than oxidative damage. Moreover, if oxidative imbalance or analytical inconsistency were occurring, simultaneous increases in total ROS, H_2_O_2_ and oxidative damage-related responses would be expected. Instead, the observed patterns were accompanied by activation of antioxidant and osmoprotective mechanisms, suggesting that MiKS formulations promoted a more controlled and balanced redox state under summer stress conditions. Similar responses have been reported in grapevine and other crops subjected to silicon applications under drought or salinity stress, where a moderate ROS burst coincided with enhanced antioxidant activity and reduced lipid peroxidation (Rizwan et al., 2015; Iqbal et al., 2021).

Among the tested formulations, MiKS 2 and MiKS 3 consistently displayed the most favorable and stable responses, combining lower ROS accumulation with balanced proline levels. These responses are consistent with previous studies reporting enhanced antioxidant capacity, improved phenolic composition and better leaf physiological performance under both single kaolin applications and MiKS treatments. Indeed, the pronounced mitigation of oxidative pressure observed with these formulations suggests that the combination of a more reflective leaf surface, reinforced cuticular properties (Pereira et al., 2025a) and moderate-to-high silicon levels creates a leaf environment less prone to excessive ROS accumulation. This mechanism is likely to be particularly relevant under heatwaves and periods of high irradiance, conditions that are expected to become increasingly frequent in warm viticultural regions (Soylemezoglu et al., 2009; Dinis et al., 2016a; Pereira et al., 2025b).

### 4.4 Antioxidant enzymatic responses

To further elucidate the mechanisms underlying MiKS-induced redox modulation, the activity of key antioxidant enzymes involved in ROS detoxification, namely superoxide dismutase (SOD) and catalase (CAT), was subsequently analyzed. SOD constitutes the first line of enzymatic defense against oxidative stress by catalyzing the dismutation of superoxide radicals (O_2_•^-^) into hydrogen peroxide (H_2_O_2_), whereas CAT plays a central role in redox homeostasis by rapidly converting H_2_O_2_ into water and oxygen, thereby preventing its accumulation and potential cytotoxic effects (Wang et al., 2018).

Beyond the physical effects of kaolin, silicon appears to play a central role in fine-tuning the enzymatic antioxidant system and shaping redox signaling dynamics. Several reviews have consistently reported that silicon enhances the activity of key antioxidant enzymes, including SOD, CAT and APX, while reducing the accumulation of H_2_O_2_ and malondialdehyde in plants exposed to drought, salinity or heat stress (Rizwan et al., 2015; Seal et al., 2018; Thakral et al., 2021). Rather than acting solely as a constitutive antioxidant booster, silicon is increasingly recognized as a modulator of redox homeostasis, capable of balancing ROS detoxification with redox signaling. In grapevine, Soylemezoglu demonstrated that silicon application altered antioxidant activity and stomatal behavior under salinity stress, pointing to a direct involvement of silicon in defense signaling pathways and water-use regulation (Soylemezoglu et al., 2009). In agreement with these observations, our results indicate that MiKS 2, and to a lesser extent MiKS 3 and MiKS 4, not only reduced H_2_O_2_ levels but were also associated with enhanced CAT activity. This pattern strongly suggests an efficient control of H_2_O_2_ homeostasis, preventing its accumulation while preserving its signaling function during stress adaptation.

Importantly, the observed reductions in SOD activity in several MiKS treatments, particularly under conditions where O_2_•^-^ levels were moderately increased, should not necessarily be interpreted as impaired antioxidant capacity. Instead, this pattern is consistent with a *redox priming* mechanism, whereby a controlled and transient accumulation of O_2_•^-^ acts as an early signaling cue, triggering downstream activation of antioxidant defenses. Under this scenario, the subsequent conversion of H_2_O_2_ is efficiently handled by CAT, preventing oxidative damage despite lower SOD activity. Such decoupling between SOD activity and O_2_•^-^ levels has been reported in plants subjected to silicon supplementation under abiotic stress and reflects a strategic shift from constitutive ROS scavenging toward regulated redox signaling and stress preparedness (Rizwan et al., 2015; Seal et al., 2018; Thakral et al., 2021). Taken together, these results support a complementary and hierarchical model of MiKS action, in which kaolin-mediated microclimate amelioration may reduce basal ROS production, while silicon can modulate enzymatic antioxidant pathways to maintain ROS at signaling-competent but non-damaging levels. This coordinated regulation of SOD, CAT and ROS species provides a mechanistic basis for the improved redox balance and stress tolerance observed under MiKS treatments, particularly in formulations with moderate-to-high silicon content.

The adjustment of the experimental design between 2023 and 2024, with the discontinuation of MiKS 1 and the introduction of MiKS 4, enabled the exploration of a broader silicon concentration gradient and provided further evidence for a dose-dependent response, as previously reported for silicon-mediated stress mitigation (Mateos-Naranjo et al., 2015; Yan et al., 2018). In 2024, particularly at E-L38, MiKS 3 and MiKS 4 induced the largest reductions in total ROS, indicating that increasing silicon availability can enhance oxidative stress mitigation up to a certain threshold. Notably, however, MiKS 2 frequently exhibited a more favorable balance between reduced H_2_O_2_ accumulation and the maintenance of proline at physiologically beneficial levels. This suggests that intermediate silicon concentrations may be sufficient to reinforce redox protection while preserving osmotic and metabolic stability. From a physiological perspective, these results support the existence of a non-linear silicon dose–response relationship, in which moderate silicon levels can promote efficient stress acclimation, whereas higher doses primarily amplify ROS attenuation without necessarily conferring additional metabolic advantages (Mostofa et al., 2021).

### 4.5 Transcriptional regulation of oxidative stress-related genes

The analysis of oxidative stress–related gene expression further supports the biochemical and physiological patterns observed in this study, indicating that MiKS formulations not only reduce ROS and H_2_O_2_ accumulation but also activate the enzymatic antioxidant machinery at the transcriptional level. Although changes in gene expression and enzymatic activity were not always strictly related, the overall trends observed for *FeSOD* and *CAT* expression were broadly consistent with the modulation of SOD and CAT activities across treatments and phenological stages. This partial decoupling likely reflects post-transcriptional regulation and enzyme turnover dynamics, which are common features of antioxidant responses under fluctuating field conditions, where rapid redox adjustments are required (Van Ruyskensvelde et al., 2018). It will be also important to refer that the different patterns observed between *FeSOD* gene expression and total SOD enzymatic activity may result from the specific detection of a single SOD isoforme (*FeSOD*) by qPCR, whereas the enzymatic assay reflects the combined activity of multiple SOD isoforms. Also, will be important to refer that post-transcriptional and temporal differences between transcription and enzyme activation may contribute to this discrepancy. The overall tendency toward *FeSOD* and *CAT* up-regulation across years and phenological stages is consistent with an enhanced capacity for O_2_•^-^ and H_2_O_2_ detoxification induced by the combined application of kaolin and silicon. For *FeSOD*, a pronounced transcriptional induction was observed in MiKS 3-treated plants, particularly at E-L35 in 2023, where this treatment showed a remarkably strong increase in expression relative to the control. This response is in agreement with the pivotal role of SOD as the first enzymatic barrier against oxidative stress and with previous reports demonstrating that silicon can stimulate *SOD* gene expression under drought, salinity or hypoxic conditions (Soylemezoglu et al., 2009; Mostofa et al., 2021). The marked induction of *FeSOD* under the above-mentioned conditions may suggest that the superoxide burst associated with environmental stress and/or MiKS application is rapidly sensed and transduced into transcriptional activation, potentially contributing to the prevention of prolonged oxidative damage (Apel and Hirt, 2004; Foyer and Noctor, 2005).

The expression pattern of *CAT* was closely aligned with changes in H_2_O_2_ content, showing pronounced induction in MiKS 3 at E-L35 in 2023 and in MiKS 2 at E-L38 in 2023 and E-L35 in 2024, conditions under which the strongest reductions in H_2_O_2_ were also recorded. Given the central role of CAT in detoxifying H_2_O_2_ generated in peroxisomes and mitochondria, and its function in completing the O_2_•^-^ → H_2_O_2_ → H_2_O detoxification cascade in coordination with SOD, the co-induction of *FeSOD* and *CAT* provides strong mechanistic support for the observed control of H_2_O_2_ levels, particularly in MiKS 2 (Soylemezoglu et al., 2009; Mostofa et al., 2021).

Importantly, the transcriptional responses can help to explain the functional differences among MiKS formulations. In 2023, MiKS 3 combined the strongest early reductions in total ROS and the highest proline accumulation at E-L35 with the most pronounced induction of *FeSOD* and *CAT*, which can indicate a rapid and robust antioxidant activation during the initial stress phase. In contrast, MiKS 2 showed its strongest transcriptional effects mainly in 2024, with elevated *CAT* expression at E-L35 and relatively higher *FeSOD* expression at E-L38, coinciding with superior H_2_O_2_ control and a more stable proline profile. This temporal shift can suggest that silicon dose influences not only the intensity but also the timing of antioxidant gene activation, helping to explain why intermediate silicon levels (4% Si) may provide a more balanced integration of redox protection and osmotic regulation. Thus, the clear and consistent transcriptional activation of *FeSOD* and *CAT* supports the conclusion that MiKS formulations stimulate the enzymatic arm of the antioxidant system, complementing the osmoprotective role of proline and the photoprotective effects of kaolin previously discussed (Dinis et al., 2016a; Mostofa et al., 2021). Together, these coordinated responses underpin the improved redox homeostasis and stress resilience observed under MiKS treatments.

The data generated in this study reinforce the view that MiKS formulations operate at multiple, interconnected levels of the grapevine oxidative stress response: (i) reducing ROS generation through kaolin-mediated modification of the leaf microclimate; (ii) promoting osmoprotection and structural stabilization via transient proline accumulation; and (iii) up-regulating key antioxidant genes such as *FeSOD* and *CAT*, thereby ensuring the efficient detoxification of O_2_•^-^ and H_2_O_2_. The integration of physiological, biochemical and molecular responses highlighted in this study supports the potential of kaolin–silicon formulations as a robust adaptive viticultural strategy under increasingly challenging climate-change scenarios (Dinis et al., 2016b; Mostofa et al., 2021).

### 4.6 Integrated antioxidant regulation and correlation analysis

The integration of physiological, biochemical and molecular responses indicates that MiKS formulations act at multiple levels of grapevine oxidative stress regulation. Besides reducing ROS accumulation, the treatments promoted transient osmoprotective responses and modulated antioxidant enzyme activity and gene expression.

The correlation analysis further supported the existence of coordinated antioxidant responses. ROS showed a strong positive correlation with CAT activity, while O_2_•^-^ was moderately positively correlated with CAT. H_2_O_2_ also showed a positive relationship with SOD activity, reflecting the integrated functioning of antioxidant pathways. In addition, the strong correlation between CAT and SOD gene expression suggests coordinated transcriptional regulation of antioxidant-related genes. Conversely, proline showed weak correlations with most oxidative and enzymatic parameters, indicating that osmoprotective responses may have operated relatively independently from the enzymatic antioxidant system.

These results reinforce the potential of kaolin–silicon formulations as sustainable tools capable of modulating grapevine redox homeostasis under increasingly variable Mediterranean climate conditions.

## 5 Conclusions

Within the context of sustainable viticulture in the Douro region, these findings have relevant agronomic and environmental implications. MiKS 2 (2% Kl + 4% Si) and, to a lesser extent, MiKS 3 (2% Kl + 6% Si) emerge as promising tools to protect ‘Touriga Franca’ during critical phenological stages, such as veraison and harvest, by mitigating leaf oxidative damage and contributing to the stabilization of photosynthetic performance. By combining the photoprotective effects of kaolin with the physiological and antioxidant benefits of silicon, these formulations may also help maintain leaf water status, improve stress acclimation, and reduce the negative impacts of heat and drought stress under Mediterranean conditions. Moreover, the use of kaolin and silicon, two low-impact and environmentally benign materials, aligns with circular economy principles and the reduction of synthetic inputs, making MiKS formulations compatible with integrated and organic viticulture systems. These results highlight the potential of kaolin–silicon-based strategies as practical and sustainable tools to enhance grapevine resilience under increasingly challenging climate conditions.

## Data Availability

The original contributions presented in the study are included in the article/supplementary material. Further inquiries can be directed to the corresponding authors.
